# Cellular Context of Panx1 Transcript Detection in Experimental Ischemic Stroke

**DOI:** 10.3390/biology15211923

**Published:** 2026-10-23

**Authors:** Yanxun Zhou, Shutong Liu, Zhihui He

**Affiliations:** 1 Medical School, Southeast University, Nanjing 210009, China; xlezi@163.com; 2 School of Medicine, Tongji University, Shanghai 200331, China; liushutong0217@gmail.com

**Keywords:** Pannexin 1, ischemic stroke, microglia, macrophages, single-cell RNA sequencing, pseudotime, neuroinflammation

## Abstract

Pannexin 1 (Panx1) participates in ATP release and inflammatory signaling, but its transcript-level distribution after ischemic stroke remains incompletely characterized. We combined a focused bibliometric analysis of 280 publications with reanalysis of GSE174574, comprising three sham and three middle cerebral artery occlusion (MCAO) mouse brain samples collected at 24 h, and a supportive tissue-level comparison using GSE58720, comprising three samples each from sham, MCAO, and MCAO-plus-vascular-endothelial-growth-factor (VEGF) groups. The curated single-cell atlas contained 58,333 cells, of which 640 had detectable Panx1 transcripts. Sample-aware comparisons used equal-weight sample fractions, exact permutation tests, and separately calculated Welch confidence intervals. The MCAO-minus-sham difference in activated microglial detection was 1.153 percentage points (95% confidence interval 0.150–2.155; exact *p* = 0.10; Benjamini–Hochberg-adjusted *p* = 0.433). Slingshot described myeloid state-space organization; its primary branch contained 10,492 cells, including 10,487 microglial cells. In bulk tissue, the Panx1 coefficient was −0.311 on the processed-matrix scale (adjusted *p* = 0.435), whereas an exploratory 16-gene myeloid/inflammatory panel had a higher mean score in MCAO (difference 1.363; exact and adjusted *p* = 0.10). Removing Panx1 from that panel preserved the direction. The results describe sparse, heterogeneous Panx1 transcript detection and accompanying inflammatory patterns, with limited between-condition inference from three samples per group. They do not establish a Panx1-specific transcriptional program, biological progression, protein induction, or channel activation.

## Simple Summary

Pannexin 1 (Panx1) is a membrane channel involved in communication between cells. We examined where its RNA was detected in a public mouse stroke dataset and compared related gene-expression patterns in a separate brain tissue dataset. Panx1 RNA was detected in only a small proportion of cells. Its distribution differed among cell types, but comparisons between three stroke and three sham samples provided limited evidence for changes within individual cell types. A descriptive analysis placed Panx1-detected cells within several myeloid transcriptional states. The separate tissue dataset showed inflammatory gene-expression patterns without a corresponding increase in Panx1 itself. These observations help identify cellular contexts for further experiments. They do not demonstrate increased Panx1 protein, channel activity, or a causal role in stroke injury.

## 1. Introduction

Pannexins (Panx) are a family of membrane proteins that form large-pore channels, comprising three isoforms: Panx1, Panx2, and Panx3 [1]. Among these, Panx1 is the most ubiquitously expressed isoform. Pannexin channels play central roles in paracrine and autocrine signaling and are critical to the control of several physiological processes, including apoptosis [2,3], blood pressure [4,5], cancer progression [6,7], mechanical allodynia [8], chronic epilepsy [9], neurological disorders and pain [10,11,12,13]. Genetic mutations of Panx1 have also been reported to cause abnormal oocyte development leading to female infertility [14]. In accordance with a broad spectrum of biological functions, Panx1 is reportedly activated by diverse physiological mechanisms [15,16,17], including signaling pathways mediated by membrane receptors [5,10,18,19,20,21,22], elevated extracellular potassium [23,24,25] or intracellular calcium levels [26], proteolytic cleavage by caspases [27,28], hypoxemia [29] and membrane stretch and voltage [7,24,26,30,31,32].

Recently, several groups resolved the cryo-EM structures of Panx channels, significantly advancing the understanding of Panx channels’ structure and their gating mechanisms [33,34,35,36,37,38,39,40,41,42].

Over the past two decades, Panx1 has been increasingly investigated in vascular biology, neuroinflammation, ischemia–reperfusion injury, immunometabolism, and multiple forms of regulated cell death. This expanding literature has underscored the broad relevance of Panx1, but it has also raised an important interpretive question: Panx1 is implicated across diverse disease models, yet its biological significance may depend strongly on the cellular and pathological context in which it is detected.

A central unresolved issue is how the biological relevance of Panx1 varies across cell types and disease-associated states and whether its detection preferentially accompanies specific inflammatory or stress-response programs. Addressing this question requires moving beyond disease-level associations toward higher-resolution evidence that identifies the cell populations in which Panx1 transcripts are detected and determines how these cells are positioned within disease-associated transcriptional states.

In this study, we first performed a focused bibliometric analysis to examine the temporal evolution of major themes in Panx1 research. Keyword patterns and trend-topic analysis showed a progression from fundamental themes involving pannexin channels, hemichannels, ATP, and purinergic signaling toward disease-related processes including ischemia, inflammation, neuroinflammation, macrophage-associated responses, mitochondrial stress, calcium signaling, and regulated cell death. Guided by this convergence of Panx1 research with ischemic and inflammatory biology, we next examined Panx1 at single-cell resolution in experimental ischemic stroke. Using a mouse middle cerebral artery occlusion (MCAO) single-cell RNA-sequencing dataset, we characterized the cell-type distribution of Panx1 transcript detection, examined its relationship with myeloid activation states and pseudotime organization, and subsequently evaluated the resulting single-cell-derived myeloid/stress signature in an independent bulk transcriptomic dataset. Together, this framework links the evolving research landscape of Panx1 with cell-state-resolved evidence in an ischemic disease context.

## 2. Materials and Methods

### 2.1. Bibliometric Data Source and Literature Screening

A bibliometric analysis was conducted using the Web of Science Core Collection (WoSCC). The literature search was completed on 4 January 2026. The topic search strategy was: TS = (“Pannexin 1” OR “Pannexin-1” OR Pannexin1 OR PANX1 OR Panx1 OR (“pannexin channel” AND Panx1)). The expanded search strategy was used to capture common spelling and naming variants of Panx1.

Original research articles and review articles published in English between 1 January 2002 and 4 January 2026 were included. Editorials, letters, meeting abstracts, and non-English records were excluded. After screening, 280 publications were included in the bibliometric analysis.

### 2.2. Keyword Analysis and Temporal Trend Analysis

To provide a focused overview of the evolving research landscape surrounding Panx1, the bibliometric analysis was restricted to keyword patterns and their temporal evolution, which were used to contextualize the subsequent transcriptomic analyses. Keywords were harmonized before analysis by merging synonymous terms and spelling variants, including Panx1/PANX1/pannexin 1, pannexin channel/pannexin channels, inflammasome/NLRP3 inflammasome, and calcium/calcium signaling.

**Figure 1:**
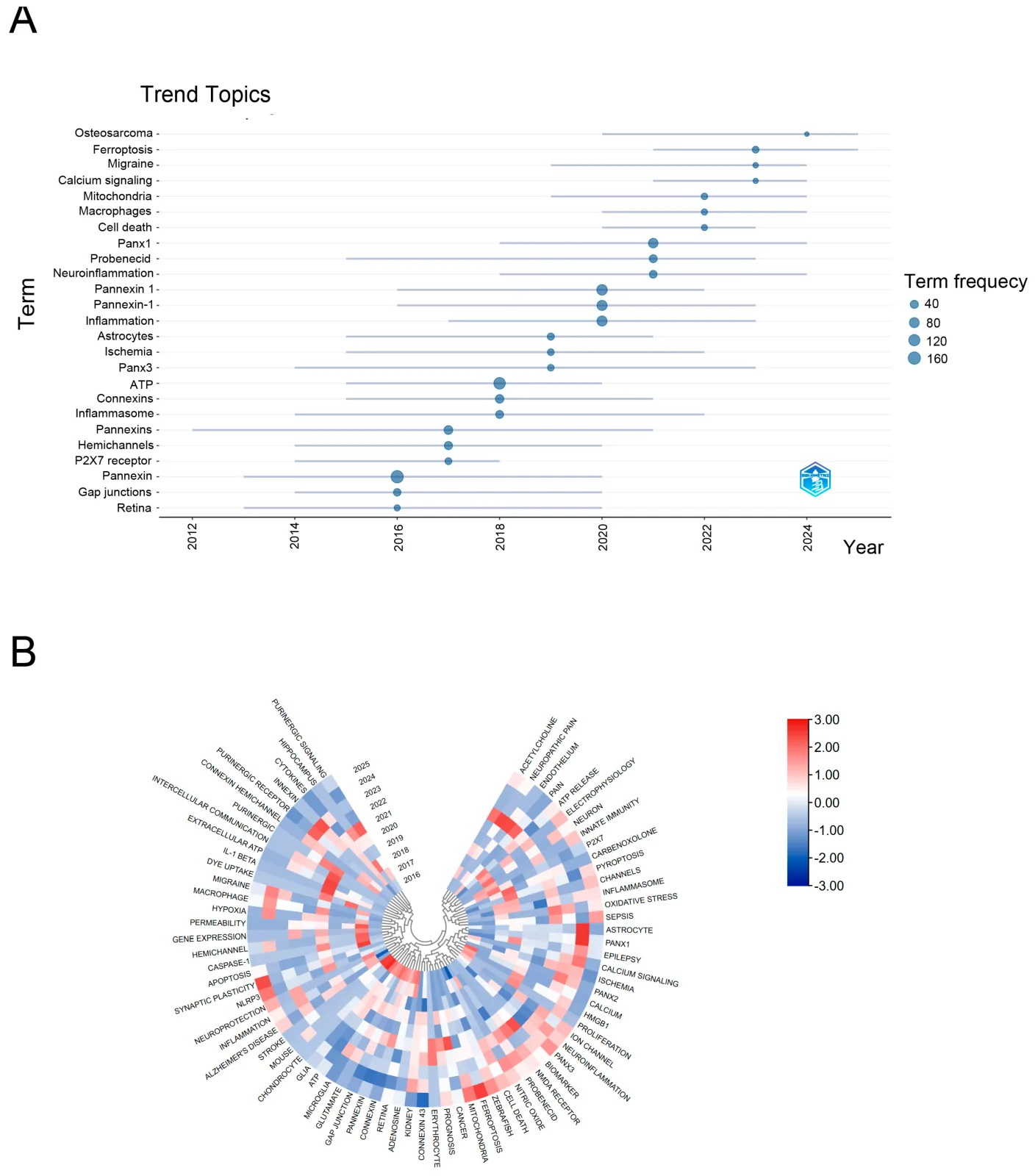
Temporal evolution of major themes in Panx1 research. (**A**) Trend-topic analysis showing the temporal distribution of major Panx1-related research terms. Horizontal lines indicate the temporal span of individual topics, and dot size represents term frequency. (**B**) Circular heatmap showing temporal patterns of major Panx1-related research terms from 2016 to 2025. Colors indicate relative temporal intensity, with red and blue representing higher and lower values, respectively. Hierarchical clustering groups terms with similar temporal patterns.

Temporal trends in research topics were analyzed using the Trend Topics function of the R software version 4.3.3 (R Core Team, Vienna, Austria). This analysis characterized the temporal distribution and evolution of frequently occurring keywords across publication years (Figure 1A).

To further investigate changes in research themes over time, a keyword–year occurrence matrix was constructed using a custom R script (Export Top500 Keyword-Year Matrices.R) implemented with the tidyverse, stringr, readr, and purrr packages. Publication years (PYs) and author keywords (DEs) were extracted from Web of Science records. Keywords were standardized through case normalization, whitespace removal, and synonym harmonization. Duplicate occurrences of the same keyword within individual publications were removed before frequency calculation. Keywords were ranked according to their overall occurrence frequencies, and the 500 most frequent keywords were initially retained. The top 100 keywords were subsequently selected for visualization.

Together, these analyses were used to characterize the shift in the Panx1 literature from fundamental channel and purinergic signaling toward ischemia-, inflammation-, and neuroinflammation-associated biological themes. The bibliometric analysis was used as a descriptive framework for identifying emerging research themes rather than as evidence of biological causality.

### 2.3. Single-Cell Dataset, Quality Control, and Integration

The discovery dataset GSE174574 comprises mouse brain single-cell RNA-seq profiles from three sham and three MCAO samples collected 24 h after surgery [43]. Raw 10x Genomics matrices were imported into Seurat using CreateSeuratObject with min.cells = 3 and min.features = 200. Cells were retained with 200–6000 detected features and mitochondrial RNA percentages of no more than 20% using the mitochondrial gene prefix mt-. Per-sample SCTransform used glmGamPoi (1.14.3) while regressing mitochondrial percentage [44,45,46]. SCT reciprocal PCA integration used 3000 integration features, 50 per-sample principal components, and dimensions 1–30 for anchor identification and integration [45]. The integrated analysis used 50 principal components and dimensions 1–30 for UMAP and nearest-neighbor construction. Resolutions 0.4, 0.6, and 0.8 were generated; the manually annotated atlas used resolution 0.6, which contained 27 graph-based clusters before cell-type annotation. The supplied workflow records contain no dedicated computational doublet-removal or ambient-RNA correction step. These limitations were retained rather than adding undocumented processing. Saved session information and the actual parameter inventory are provided in the Supplementary Methods and Supplementary Data S4–S6.

The final manually curated atlas contained 58,333 cells, including 27,216 sham cells and 31,117 MCAO cells. Major cell compartments included endothelial cells, microglia, activated microglia, monocyte/macrophages, CNS-associated macrophages, neutrophils, T/NK cells, astrocytes, oligodendrocytes, pericyte/VSMC, choroid plexus epithelial cells, ciliated ependymal cells, and ambiguous Ttr-high cells.

### 2.4. Cell-Type Annotation and Panx1 Transcript-Detection Definition

Cell types were annotated by reviewing expression summaries for curated marker panels and then applying a manual mapping of 27 clusters to 13 classes. The 13 classes are annotation categories, not the number of clusters returned by the graph-based clustering algorithm. Vascular markers included Pecam1 and Cldn5; microglial markers included P2ry12 and Tmem119; activated myeloid markers included Apoe and Lgals3; monocyte/macrophage markers included Lyz2 and Ccr2; CNS-associated macrophage markers included Mrc1 and Pf4; and neutrophil markers included Retnlg and Csf3r. Complete marker panels, cluster-level expression summaries, and the manual mapping are provided in Supplementary Data S4. Ambiguous Ttr-high cells were retained as a separate class. This annotation procedure did not involve a documented all-gene FindAllMarkers analysis. Panx1 transcript detection was defined as raw RNA Panx1 UMI > 0. Because detection was sparse, analyses used Panx1-detected and Panx1-not-detected categories.

**Figure 2:**
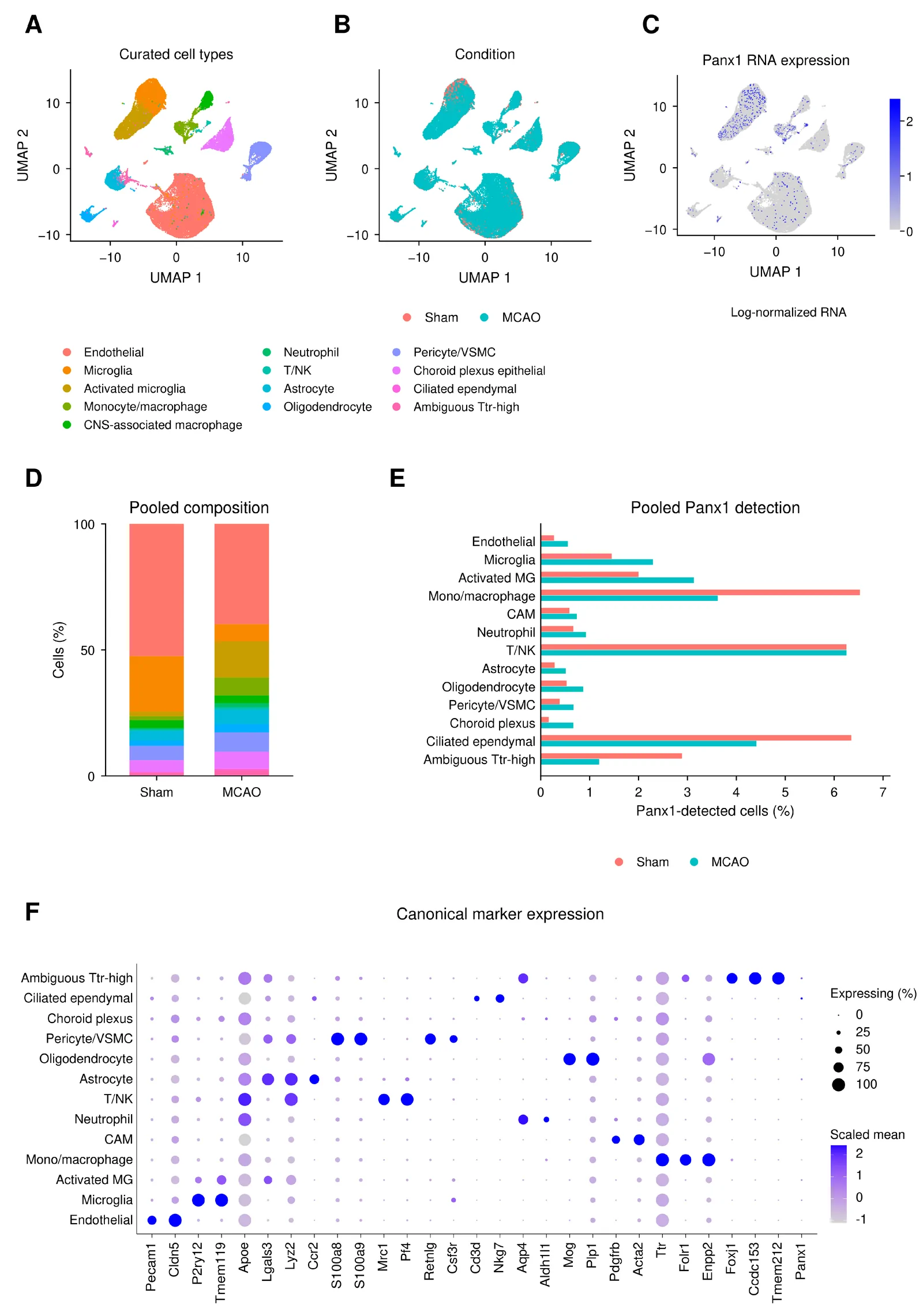
Panx1 transcript detection across the mouse brain atlas. (**A**) Existing UMAP coordinates of 58,333 cells, colored by the 13 manually curated classes. (**B**) The same embedding colored by condition. (**C**) Panx1 RNA LogNormalize expression; the color scale is normalized expression, whereas binary detection throughout this study is defined as raw RNA UMI > 0. (**D**) Pooled cell-type composition within each condition. (**E**) Pooled Panx1-detected fraction within each cell type and condition. Panels (**D**,**E**) are descriptive cell summaries; the biological replication is three sham and three MCAO samples, with sample-level inference in Supplementary Data S1. (**F**) Canonical-marker DotPlot. Dot size represents the percentage of cells expressing each gene, and color represents Seurat-scaled average expression. The ambiguous Ttr-high class remains visible. UMAP position and marker expression do not establish lineage relationships or Panx1 channel activity.

This binary definition measures transcript detection and does not measure Panx1 protein abundance, membrane localization, channel opening, or functional activation. Continuous RNA expression displayed in Figure 2C was obtained from the RNA LogNormalize data layer; it is distinct from the raw-count threshold used to define detection.

To describe technical factors associated with transcript detection, we summarized the recorded RNA-assay UMI total (nCount_RNA) and detected-gene count (nFea-ture_RNA) by biological sample, condition, cell type, and Panx1 detection status. Each stratum is reported with its cell count, median, first and third quartiles, mean, and standard deviation in Supplementary Data S1. All 156 sample-by-cell-type-by-status combinations were retained, including empty detected strata with count zero and missing QC summaries. Nonempty strata with fewer than ten cells were flagged rather than excluded. The metrics were taken from the unchanged final RNA-assay metadata, not SCT-corrected counts or integrated expression. These metrics describe captured RNA in the retained assay, not sequencing-read counts or directly measured cellular RNA content. Pooled summaries and within-sample status comparisons were descriptive; they did not provide depth-adjusted condition effects. No cell-level hypothesis test was applied to these QC comparisons.

### 2.5. Myeloid Modules and Transcriptional State-Space Organization

The focused myeloid compartment comprised microglia, activated microglia, CNS-associated macrophages, monocyte/macrophages, and neutrophils. It retained the whole-atlas UMAP coordinates and resolution-0.6 cluster labels. Sample-level Panx1-detected fractions used the number of cells in the corresponding sample and cell type as the denominator; cell-type proportions used either all atlas cells or all myeloid cells in that sample, as specified in Section 2.7.

**Figure 3:**
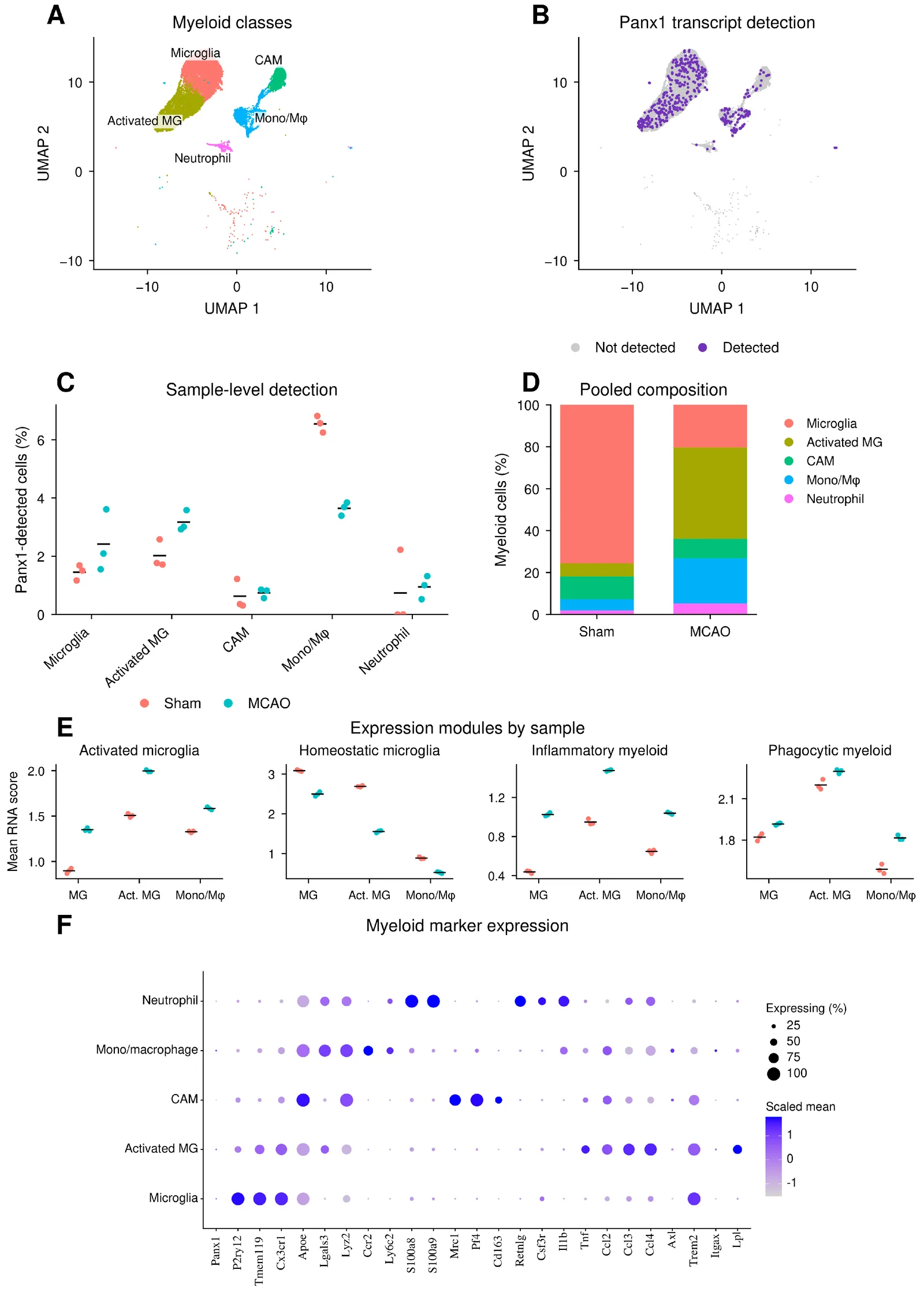
Panx1 transcript detection and expression modules in five myeloid classes. (**A**) The 18,203-cell myeloid subset shown in its existing atlas UMAP coordinates. (**B**) Binary Panx1 transcript-detection overlay; gray indicates not detected, and purple indicates raw RNA UMI > 0. (**C**) Detection fractions for each biological sample, with three samples per condition; short horizontal lines indicate unweighted sample means. Effect estimates in percentage points, separately calculated Welch 95% confidence intervals, exact *p* values, and BH-adjusted *p* values are provided in Supplementary Data S1. (**D**) Pooled myeloid composition within condition. (**E**) Sample-level mean scores for four manually specified modules in Microglia, activated microglia, and monocyte/macrophages; points are samples, and horizontal lines are condition means. Scores average available genes in the RNA LogNormalize data layer. (**F**) Myeloid marker DotPlot; size denotes percentage expressing, and color denotes Seurat-scaled average expression. CAM denotes CNS-associated macrophages. These displays describe cell composition, transcript detection, and expression patterns. They do not identify a separate Panx1 lineage or establish a Panx1-dependent activation process.

**Figure 4:**
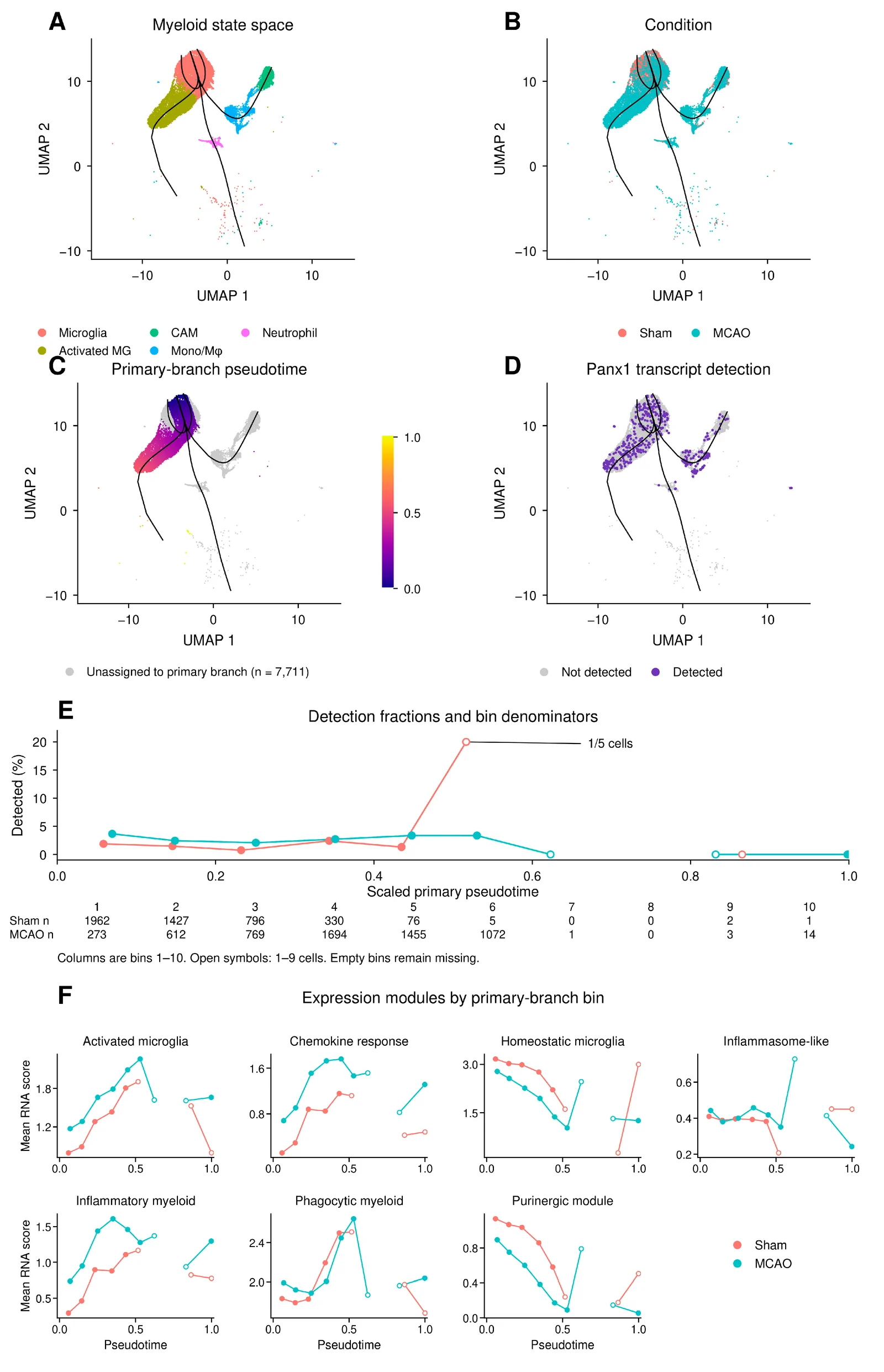
Panx1 transcript detection within descriptive myeloid state-space organization. (**A**) Existing myeloid UMAP coordinates colored by cell type, with the five retained Slingshot curves. (**B**) The same coordinates colored by condition. (**C**) Primary-branch pseudotime scaled to 0–1 for 10,492 cells; 7711 cells unassigned to that branch remain gray. Curves depict all five branches, while the color scale refers only to Lineage2. (**D**) Panx1 transcript-detection overlay using the same gray/purple definition as Figure 3B. (**E**) Condition-pooled Panx1-detected fractions across ten primary-branch bins. Empty bins are missing, line segments do not bridge them, and open symbols flag nonempty bins with fewer than ten cells. The denominator display reports the cell counts by condition; sample-specific support is provided in Figure S1 and Supplementary Data S3. (**F**) Mean expression-module scores in the same condition/bin groups, with missing bins left unconnected and low-cell bins flagged. Root selection used condition information. Neither the curve geometry nor pseudotime denotes elapsed time, biological progression, cross-lineage conversion, or Panx1-dependent causality.

Gene modules were specified manually in the analysis scripts to summarize homeostatic microglial, activated microglial, inflammatory myeloid, phagocytic myeloid, chemokine-response, inflammasome-like, and purinergic programs. Published work on microglial identity and disease-related transcriptional phenotypes provides biological context for several of these genes [47,48,49], but the original gene-by-gene selection record was unavailable. These references therefore provide contextual support rather than a documented derivation of the exact panels. Per-cell module scores were the arithmetic mean of available genes in the RNA LogNormalize data layer; no matched-control-gene subtraction was used. The four modules displayed in Figure 3E and all seven modules in Figure 4F are listed with requested, available, and unavailable genes in Supplementary Data S4. Clec7a was absent from the single-cell phagocytic module input, which left seven of the eight requested genes.

Slingshot was applied to the 18,203-cell myeloid subset using the existing UMAP coordinates and cluster labels [50]. During revision, a controlled rerun using the same coordinates, cluster labels, and root-selection rule reproduced the available pseudotime values, bin assignments, and exported curve coordinates within the stated numerical tolerance (Supplementary Methods, Section S6). Figure preparation used these retained outputs without further trajectory fitting. Candidate root clusters were annotated as Microglia and contained at least 500 cells. They were ordered by ascending MCAO fraction, then descending mean homeostatic module score, then descending cell count, selecting cluster 2. Because this rule used condition information, orientation was not an independent test of disease direction. Five branches were retained, and the branch with the largest number of non-missing pseudotimes was selected as primary (Lineage2; clusters 2–19–4). Its pseudotime was rescaled to 0–1 and divided into ten equal-width intervals. Cells with missing primary-branch pseudotime were excluded from these bin summaries and remained explicitly unassigned in the display. Bin summaries pooled cells within condition; sample-specific denominators and branch contributions are provided in Supplementary Data S3 and Figure S1. Empty bins were left missing, and bins with fewer than ten cells were flagged descriptively without changing inclusion rules. The analysis describes transcriptional state-space organization across distinct myeloid populations; it does not estimate elapsed time, cell fate, or transitions between these populations.

### 2.6. Supportive Bulk Tissue Comparison

GSE58720 is an independent mouse brain microarray dataset containing three sham, three MCAO, and three MCAO-plus-VEGF samples. The source study examined VEGF-related blood–brain barrier changes after 90 min transient MCAO. Recombinant VEGF-A165 was administered into the lateral ventricle 3 h after reperfusion, and peri-infarct tissue was sampled at 24 h [51]. The VEGF group was retained as an existing intervention group, without treating it as a Panx1-positive or Panx1-negative control or assuming a higher panel score. GPL10787 annotation was used to map probes to gene symbols, with the highest-mean probe across all nine samples retained for each symbol. This produced 24,163 genes. The supplied processed expression matrix was analyzed without an additional log transformation. Because the upstream logarithm base was not established by the available preprocessing record, coefficients are reported on the processed-matrix scale rather than converted into fold changes.

The bulk analysis used a manually specified exploratory 16-gene myeloid/inflammatory panel containing Panx1, together with six related modules. The panel was not derived by sample-aware differential-expression analysis of Panx1-detected versus non-detected cells. Complete definitions and scoring inputs are provided in Supplementary Data S4; 15 of its 16 genes also occur in at least one related bulk module. Each gene was standardized across the nine samples by subtracting its mean and dividing by its sample standard deviation, and each panel or module score was the arithmetic mean of its available gene z-scores. Prespecified sensitivity summaries removed Panx1 from the two panels containing it or standardized genes across the six sham/MCAO samples. These computations assess score dependence on the chosen inputs and do not represent a biological Panx1 knockout.

Gene-level comparisons used limma with a no-intercept three-group design, contrasts for MCAO minus sham and MCAO plus VEGF minus MCAO, and empirical Bayes moderation [52]. We report the estimated coefficient, moderated 95% confidence interval, *p* value, and Benjamini–Hochberg (BH) adjustment across all 24,163 genes separately for each contrast. Complete gene-level results, panel scores, and sensitivity summaries are provided in Supplementary Data S2. Module-level tests are described in Section 2.7.

### 2.7. Sample-Aware Statistical Analysis

Biological samples were the independent units for single-cell condition comparisons (three sham and three MCAO samples, treated as unpaired). For each sample, we calculated Panx1-detected fractions within 13 cell types, proportions of the 13 cell types within the full atlas, and proportions of five myeloid classes within the myeloid subset. Cells were aggregated to these sample-level summaries before testing, and condition labels were permuted at the whole-sample level rather than across individual cells. Supplementary Data S1 reports detected-cell and total-cell counts for every sample and annotated class. The primary effect was the equal-weight MCAO-minus-sham difference in sample fractions, reported in percentage points. Two-sided exact permutation *p* values enumerated all 20 allocations of three of the six samples to each condition. Welch 95% confidence intervals were calculated separately as approximate uncertainty summaries; they are not inversions of the permutation test. BH adjustment used the prespecified families of 13, 13, and five comparisons. Leave-one-sample-out effects were retained as sensitivity summaries. With this design, the minimum two-sided exact *p* value is 0.10.

For each cell type and each sample, a 2 × 2 table cross-classified target versus reference cells and Panx1-detected versus not detected. The sample odds ratio was ad/bc, comparing each of the 13 types with all other 12 types or each of the five myeloid classes with the other four myeloid classes. Within each condition, the unweighted mean of three log odds ratios was compared with zero by a one-sample t test with two degrees of freedom; exponentiation yielded a geometric mean odds ratio and its 95% confidence interval. A zero cell in any contributing contingency table resulted in a not-estimable (NE) summary, without a continuity correction. No contributing sample was excluded because of a zero count; five of the 36 planned summaries could not be estimated. BH and BY adjustments used the full planned families of 26 and ten condition/type contrasts, including the planned NE entries in the family size. These odds ratios are within-condition comparisons, not MCAO-versus-sham effects. These exploratory odds ratios are within-condition comparisons, not MCAO-versus-sham effects.

Bulk module comparisons used the difference in sample means, two-sided exact permutation *p* values over the 20 three-versus-three allocations for each contrast, and separately calculated Welch 95% intervals. BH adjustment was applied across the seven panel/module comparisons separately for each contrast. Scores were calculated once from the specified nine-sample standardization before label permutation. Pooled-cell plots and pseudotime-bin summaries were descriptive; cells, branches, and bins were not treated as independent biological replicates. No additional inferential test was applied to trajectory geometry or spatial randomness.

## 3. Results

### 3.1. Bibliometric Analysis Highlights an Emerging Intersection of Panx1 with Ischemic and Inflammatory Research

To identify evolving biological themes in Panx1 research, we examined the temporal distribution of high-frequency keywords. The circular keyword heatmap revealed that Panx1-related studies encompass a broad range of processes, including ATP and purinergic signaling, hemichannel activity, inflammation, ischemia, and neurological disorders (Figure 1A). Over time, the thematic landscape expanded toward inflammation- and stress-related processes, with terms such as neuroinflammation, macrophage, inflammasome, oxidative stress, mitochondria, cell death, ferroptosis, and calcium signaling becoming increasingly represented.

Trend-topic analysis further illustrated this transition (Figure 1B). Earlier research was largely centered on fundamental aspects of pannexin biology, including gap junctions, hemichannels, P2X7 receptor signaling, and ATP-related processes. These themes were subsequently accompanied by increasing attention to inflammation, astrocytes, ischemia, and neuroinflammation, followed more recently by macrophages, mitochondria, cell death, calcium signaling, and ferroptosis. Together, these temporal patterns point to a growing intersection between Panx1 research and ischemia-associated inflammatory and myeloid biology.

This convergence prompted us to examine Panx1 in a disease context in which ischemic injury and myeloid responses are closely intertwined. We therefore turned to a mouse middle cerebral artery occlusion (MCAO) single-cell RNA-sequencing dataset to determine which brain cell populations contain detectable Panx1 transcripts after ischemic stroke and whether their distribution differs between sham and MCAO conditions.

### 3.2. Panx1 Transcript Detection Is Sparse but Cell-Type-Specific in the Ischemic Microenvironment

The manually curated atlas contained 58,333 cells from six biological samples. The UMAP displays the major vascular, glial, immune, stromal, epithelial, and ambiguous Ttr-high compartments and their condition labels (Figure 2A,B). Panx1 transcripts were detected in 640 cells: 210 of 27,216 sham cells and 430 of 31,117 MCAO cells (Figure 2C,E). These pooled counts describe the atlas and do not constitute an independent test of a condition effect. Across the 13 cell types, sample-level MCAO-minus-sham detection fraction differences were evaluated using three samples per condition; all exact *p* values were at least 0.10, with corresponding uncertainty and BH-adjusted values reported in Supplementary Data S1.

Of the 640 Panx1-detected cells, 619 (96.72%) had one raw Panx1 UMI and 21 had two. Across the atlas, detected cells had a median RNA UMI total of 4315.5 (interquartile range 2674.75–6349.75), compared with 2672 (2071–3964) in the 57,693 non-detected cells. Median detected-gene counts were 1724.5 (1309–2254.5) and 1323 (1113–1681), respectively. Both statuses were observed in 71 of the 78 sample-by-cell-type strata; the other seven contained no detected cells. The detected group had the higher median UMI total in 57 of these 71 strata and the higher median gene count in 57 strata. These are separate descriptive tallies, not independent hypothesis tests. The full stratified summaries are reported in Supplementary Data S1 and indicate that detection status should be interpreted alongside captured-RNA metrics. Figure S2 displays the sample-level summaries and within-sample, within-cell-type median ratios.

Endothelial cells formed the largest compartment, and pooled cell-type proportions differed between conditions (Figure 2D). Complete sample-level proportion comparisons are reported separately from detection-fraction comparisons in Supplementary Data S1. Within the MCAO group, the sample-based geometric mean odds ratio for Panx1 detection in activated microglia relative to the other 12 cell types was 2.934 (95% CI 2.804–3.070; *p* = 0.000095; BH-adjusted *p* = 0.002482; Benjamini–Yekutieli-adjusted *p* = 0.009567). This within-condition contrast does not estimate the effect of MCAO on activated microglia. The marker DotPlot provides the expression context for the manual annotations, including the retained ambiguous compartment (Figure 2F).

### 3.3. Sample-Level Detection Comparisons in the Myeloid Compartment

The myeloid subset contained 18,203 cells spanning five annotated classes (Figure 3A). Pooled activated microglial counts were 500 in sham and 4470 in MCAO, and monocyte/macrophage counts were 429 and 2210, respectively; the Microglia class had a lower relative representation in MCAO (Figure 3D). These count and composition differences describe the sampled populations. Sample-level composition comparisons are provided in Supplementary Data S1 and did not yield an exact *p* value below 0.10.

Activated microglia contained 10 Panx1-detected cells in sham and 140 in MCAO, while monocyte/macrophages contained 28 and 80, respectively (Figure 3B,C). Absolute detected-cell counts and within-type detection fractions gave different perspectives. For monocyte/macrophages, these counts corresponded to pooled detection fractions of 28/429 (6.53%) in sham and 80/2210 (3.62%) in MCAO: more detected cells were captured despite a lower within-type detection fraction. The equal-weight sample means were 6.546% and 3.643%, respectively, and were kept distinct from the pooled fractions. The sample-level MCAO-minus-sham difference was +1.153 percentage points for activated microglia (Welch 95% CI 0.150–2.155; exact *p* = 0.10; BH-adjusted *p* = 0.433), +0.964 for Microglia (−1.503–3.432; *p* = 0.20; adjusted *p* = 0.520), and −2.902 for monocyte/macrophages (−3.500 to −2.305; *p* = 0.10; adjusted *p* = 0.433). The separate approximate confidence intervals do not override the exact primary tests. Within MCAO, activated microglia relative to the other four myeloid classes had an odds ratio of 1.304 (95% CI 1.034–1.645; *p* = 0.038996; BH-adjusted *p* = 0.087123; BY-adjusted *p* = 0.255180). Thus, the reference population materially affected the odds-ratio comparison. Module and marker summaries describe the accompanying myeloid expression patterns (Figure 3E,F), without testing a Panx1-specific program.

### 3.4. Descriptive Organization of Panx1-Detected Cells in Myeloid State Space

Slingshot produced five branches within the existing myeloid UMAP, with terminal clusters annotated as CNS-associated macrophages, activated microglia, Microglia, neutrophils, and Microglia (Figure 4A,B). Branch membership overlapped: 5787 cells had non-missing pseudotime in more than one branch. The selected primary branch contained 10,492 cells, including 5530 Microglia, 4957 activated microglia, three CNS-associated macrophages, and two monocyte/macrophages. It covered 57.64% of the myeloid subset; the other 7711 cells had no primary-branch pseudotime (Figure 4C). These branches are descriptive comparisons across the observed transcriptional space, not evidence that one myeloid lineage differentiates into another. All six samples contributed to the primary branch, with individual shares of 13.29–20.98% (Figure S1; Supplementary Data S3).

The primary branch contained 74 Panx1-detected cells among 4599 sham cells and 172 among 5893 MCAO cells (Figure 4D). These are pooled descriptive counts. Detection fractions varied across bins, but some bins had very limited support: the apparent 20% sham value in Bin6 represented one detected cell among five, distributed across three samples with denominators of one, three, and one. Of the 60 sample-by-bin combinations, 15 were empty and 12 contained between one and nine cells. Accordingly, the detection and module-score displays were interpreted together with their denominators (Figure 4E,F and Figure S1). We did not test whether the spatial distribution was random, infer a monotonic Panx1 response, or treat these cross-sectional coordinates as temporal progression.

### 3.5. Supportive Tissue-Level Comparison in GSE58720

**Figure 5:**
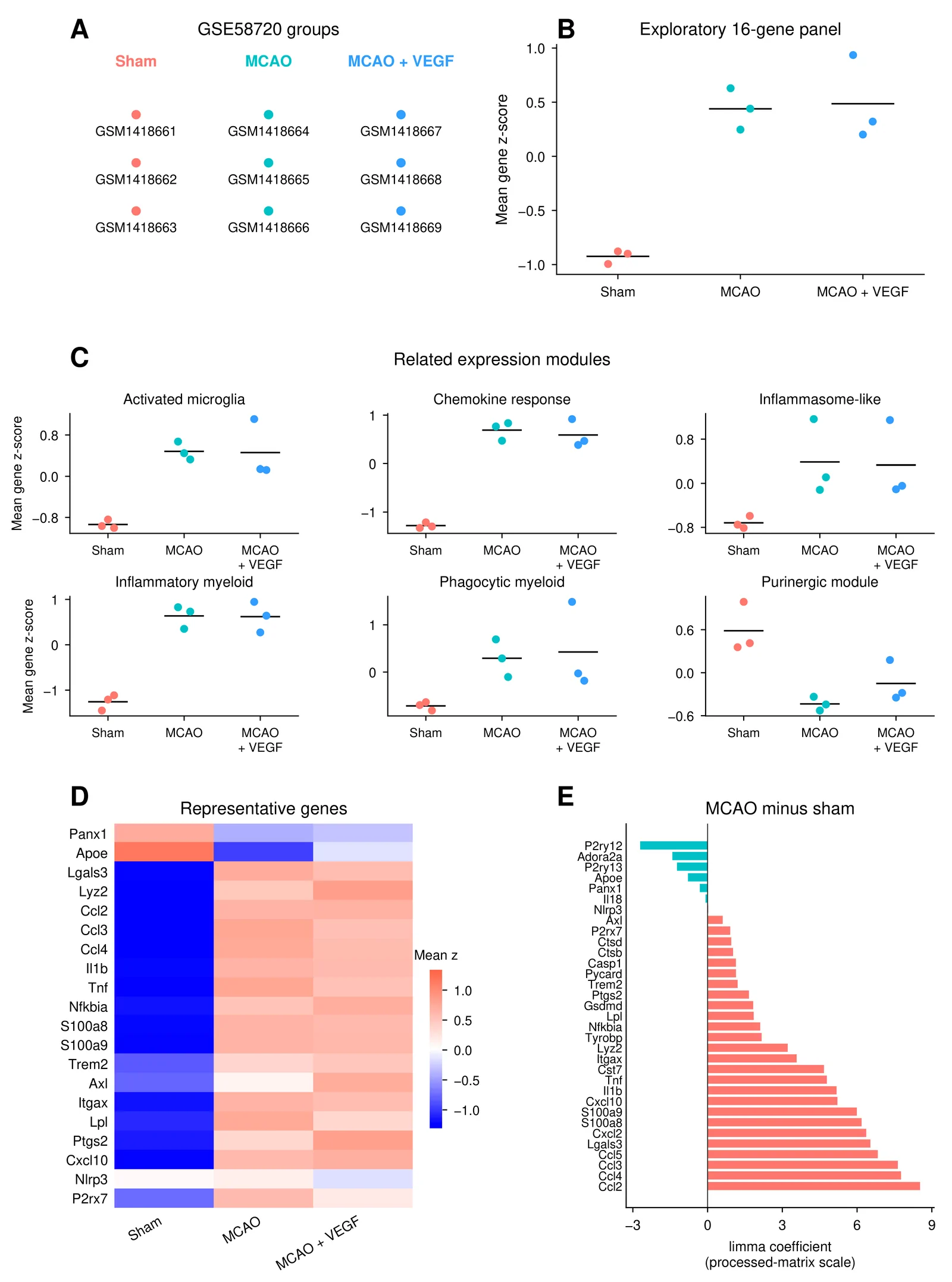
Supportive bulk tissue comparison in GSE58720. (**A**) The three groups and nine distinct array samples. MCAO plus VEGF is an existing intervention group, without a predefined Panx1-positive or Panx1-negative control role. (**B**) Sample scores for the exploratory 16-gene myeloid/inflammatory panel containing Panx1. (**C**) Six related modules. In B and C, genes were standardized across all nine samples and averaged within each module; points denote individual samples, and short lines denote unweighted group means. Exact permutation *p* values and separately calculated Welch confidence intervals are reported in Supplementary Data S2. (**D**) Group means of gene-wise z-scores for 20 representative genes, using the original gene set and order. (**E**) MCAO-minus-sham limma coefficients for the 33-gene module union, on the processed-matrix scale.Salmon-colored bars (“Positive”) indicate positive coefficients, corresponding to higher estimated expression in MCAO than in sham; teal bars (“Negative”) indicate negative coefficients, corre-sponding to lower estimated expression. “Direction only” indicates that bar colors represent co-efficient direction, not statistical significance. Full statistics include gene-wise *p* values and BH adjustment over all 24,163 genes. These tissue-level data do not identify cellular origin, establish a Panx1-specific program, or measure Panx1 protein or channel activity.

The GSE58720 comparison included three arrays per group (Figure 5A). The exploratory 16-gene myeloid/inflammatory panel had a higher sample mean in MCAO than in sham, with a difference of 1.363 mean gene z-score units (Welch 95% CI 0.938–1.788; exact *p* = 0.10; BH-adjusted *p* = 0.10; Figure 5B). The six related module differences ranged from −1.021 for the purinergic module to +1.970 for the chemokine response module; all had exact and adjusted *p* values of 0.10 (Figure 5C; Supplementary Data S2). These directional summaries are compatible with a general injury-related expression context, but their overlapping genes and small sample size limit independent validation claims.

The representative-gene heatmap and module-union coefficients showed heterogeneous gene-level directions (Figure 5D,E). Panx1 itself had an MCAO-minus-sham coefficient of −0.310891 on the processed-matrix scale (moderated 95% CI −0.832594 to 0.210811; *p* = 0.205857; BH-adjusted *p* = 0.434916). For MCAO plus VEGF minus MCAO, its coefficient was 0.032844 (−0.488858 to 0.554547; *p* = 0.887806; adjusted *p* = 0.999935). The seven panel/module comparisons for that intervention contrast had exact *p* values of 0.20–1.00, adjusted *p* values of 1.00, and Welch intervals spanning zero. Removing Panx1 from the 16-gene panel retained the MCAO-minus-sham direction, changing the difference from 1.362690 to 1.529337; the purinergic-module difference changed from −1.021003 to −0.991997. The six-sample standardization sensitivity also retained all seven disease-contrast directions. These results support a descriptive inflammatory/myeloid context, with no evidence here that the aggregate scores specifically reflect Panx1 induction or a consistent VEGF effect.

## 4. Discussion

This study combines a focused bibliometric description of Panx1 research themes with single-cell transcript detection, myeloid state-space summaries, and an independent bulk tissue comparison in experimental ischemic stroke. The literature analysis contextualized the focus on ischemia and inflammation. The transcriptomic analyses provide descriptive cellular and tissue-level observations, with explicit sample-level statistics and limitations on mechanistic interpretation.

Panx1 transcript detection was sparse across the atlas. Although the pooled proportion was numerically higher in MCAO, sample-level comparisons within the 13 annotated cell types did not attain an exact *p* value below 0.10. Within-condition odds ratios answered a different question: how detection in a selected cell type compared with the remaining cells in the same samples. The difference between the activated microglial odds ratios using all other cell types and using only other myeloid classes illustrates this dependence on the reference population. Such contrasts describe relative detection patterns; they do not establish stroke-induced Panx1 upregulation.

The myeloid compartment showed differences in the sampled composition and accompanying expression modules. More activated microglial and monocyte/macrophage cells were captured in MCAO, yet the monocyte/macrophage within-type Panx1-detection fraction was lower. Counts of detected cells must therefore be interpreted alongside both cell-type abundance and within-type detection denominators. The module summaries were manually specified descriptive scores and were not derived from a sample-aware comparison of Panx1-detected and non-detected cells. They cannot establish that Panx1 drives or uniquely identifies the associated expression patterns.

The annotation granularity differs from that of the source atlas. Zheng et al. reported 17 principal brain groups in their published whole-brain analysis of 58,528 cells using Seurat 3 and t-SNE [43]. Our retained atlas contains 58,333 cells processed with SCT reciprocal PCA integration, and its 27 resolution-0.6 clusters were assigned to 13 classes. For example, seven clusters were grouped as Endothelial and two as Pericyte_VSMC, whereas activated microglia were retained separately from the Microglia class. Three clusters with ambiguous Ttr-high expression remained explicitly labeled. No single parameter was isolated as the cause of these differences. These are different analytical and annotation resolutions, not a demonstrated one-to-one correspondence between the two atlases. Aggregation can mask subtype-specific detection patterns, and altered class boundaries change detection denominators. Accordingly, the results apply to the reported classes and do not establish stability to every alternative clustering or annotation scheme.

The QC summaries also identify an important interpretive constraint: Panx1-detected cells generally had higher captured-RNA totals and detected-gene counts, including within many sample-by-cell-type strata. These associations cannot distinguish sequencing depth, capture efficiency, and cellular RNA content from biological differences. SCTransform was used for normalization and integration, but the binary outcome was still defined from raw Panx1 UMI counts; normalization of the clustering input does not make that raw-count threshold depth-independent. Sample-level aggregation addresses biological replication, not technical confounding of detection. The condition estimates were not adjusted for these captured-RNA metrics. We therefore interpret the condition contrasts as differences in observed transcript detection rather than evidence of depth-independent Panx1 induction.

The Slingshot analysis offers a descriptive view of the observed myeloid state space. Its primary branch was almost entirely microglial, while other branches included biologically distinct macrophage and neutrophil populations. The curves do not demonstrate transitions between those populations. Root orientation incorporated the MCAO fraction, and the data came from a single post-surgery time point. Moreover, sparse bins, including a sham estimate based on one of five cells, limit interpretation of apparent local changes. The branch memberships, sample contributions, and denominators are therefore integral to the interpretation rather than supplementary evidence of progression.

The GSE58720 analysis adds an independent tissue-level comparison with a similar ischemic-injury context. Several inflammatory and myeloid scores were directionally higher in MCAO, but all module-level exact tests had a minimum attainable two-sided *p* value of 0.10 with three samples per group. Panx1 itself did not show concordant induction, and removing it from the two relevant panels retained the score directions. Because genes overlap between modules and tissue measurements cannot identify the cell of origin, these findings are most compatible with a broad injury-related expression pattern. The VEGF group was included to describe the original intervention context; its results do not provide a Panx1-specific control or demonstrate a VEGF-mediated rescue.

Panx1 transcript detection, protein localization, and channel function are separate levels of evidence. Channel output also depends on assembly, membrane localization, and gating, which were not measured here. Sparse RNA detection and the bulk coefficient therefore cannot establish either increased or decreased channel activity. Direct measurements and cell-type-specific perturbations are needed to determine whether Panx1 contributes to the myeloid states described in these datasets.

Previous studies provide relevant protein and functional context, with important differences in models and cell types. Good et al. reported Panx1 immunostaining in neurons and cerebral vascular cells, including control arteries. Their endothelial-specific deletion experiments examined inflammation and injury after 90 min of MCAO followed by 24 h of reperfusion [53]. Freitas-Andrade et al. assessed cortical Panx1 by Western blot four days after permanent distal MCAO and reported sex-dependent protection from Panx1 loss or blockade. Protein levels were overall higher in females than in males (sex main effect, *p* = 0.037), with no significant main effect of cortical side (ipsilateral versus contralateral, *p* = 0.369) [54]. This sex comparison does not establish ischemia-induced protein upregulation in females. Neither study establishes Panx1 protein induction in the activated microglia represented in our dataset. Mousseau et al. provide functional evidence involving spinal microglial Panx1 in a joint-pain model [8], which supplies context but does not validate a brain stroke protein change. This model-specific evidence provides context but does not constitute matched proteomic, immunohistochemical, or channel-activity validation of our transcriptomic findings.

These observations identify testable cellular contexts for future Panx1 experiments. Such studies could combine cell-type-resolved protein localization, channel-activity assays, and genetic perturbation at defined times after ischemia. The exploratory gene panels may help describe the accompanying inflammatory response, but their association with that response should not be assumed to imply direct Panx1 regulation. The present data do not establish a clinical biomarker or a therapeutic treatment effect.

Several limitations constrain the conclusions. Three biological samples per condition provide limited precision and restrict the resolution of exact permutation tests; a *p* value above a conventional threshold does not demonstrate the absence of an effect. The odds-ratio summaries additionally rely on the distribution of three sample log odds ratios, and comparisons with undefined sample ratios were retained as not estimable. Transcript dropout, differences in capture, and the lack of a documented dedicated doublet or ambient-RNA correction can affect detection and annotation. A single time point and condition-informed root selection prevent temporal or independent disease-direction interpretation of the state-space analysis. The original gene-selection record was unavailable, and overlapping modules provide limited independent information. The bulk data cannot resolve cellular origin, and the upstream logarithm base remained unconfirmed. Matched protein and functional assays were not available. For the bibliometric component, the raw export, harmonization dictionary, and complete plotting-parameter record were not available for independent reconstruction during revision; its count and temporal patterns remain descriptive.

The resulting framework is hypothesis-generating: it identifies where Panx1 transcripts were detected, describes the sampled myeloid states, and places those observations alongside tissue-level expression patterns. Its value is in specifying the populations and measurements for further experiments while retaining the uncertainty imposed by the available biological replication.

## 5. Conclusions

Panx1 transcripts were sparsely detected across the mouse ischemic brain atlas, with heterogeneous distributions among annotated cell types and myeloid states. Sample-level between-condition comparisons remained limited by three samples per group. Slingshot provided descriptive state-space positioning, without demonstrating biological progression or cross-lineage conversion. The independent bulk dataset showed inflammatory/myeloid expression patterns without concordant Panx1 induction or evidence of a Panx1-specific program. These results motivate targeted protein and functional studies in defined cellular contexts.

## Data Availability

The bibliometric dataset was derived from the Web of Science Core Collection using the search strategy described above; the original export and full harmonization record were not available for independent reconstruction during revision. The transcriptomic datasets are available from GEO under GSE174574 and GSE58720. The supplementary package provides sample-level counts, statistics, and captured-RNA QC summaries (S1), full bulk comparisons and score summaries (S2), descriptive branch and bin information (S3), module definitions and annotation evidence (S4), figure-source and software records (S5), and the supplied analysis scripts (S6).

## Supplementary Materials

The following supporting information can be downloaded at: https://www.mdpi.com/article/10.3390/biology15211923/s1, Supplementary Methods; Figure S1: Sample and bin support; Figure S2: Captured-RNA quality-control summaries by Panx1 transcript-detection status.

## Abbreviations

The following abbreviations are used in this manuscript:

ATP	adenosine triphosphate
CNS	central nervous system
GEO	Gene Expression Omnibus
MCAO	middle cerebral artery occlusion
Panx	pannexin
Panx1	pannexin 1
PCA	principal component analysis
RNA-seq	RNA sequencing
SCT	SCTransform
scRNA-seq	single-cell RNA sequencing
UMAP	Uniform Manifold Approximation and Projection
UMI	unique molecular identifier
VEGF	vascular endothelial growth factor
VSMC	vascular smooth muscle cell
WoSCC	Web of Science Core Collection
