# Supplementary material for: Cellular Context of Panx1 Transcript Detection in Experimental Ischemic Stroke
Source: Biology (Basel). 2026 Oct 23;15(21):1923. doi: 10.3390/biology15211923 (PMC13762306; doi:10.3390/biology15211923)
Supplement: Supplementary file 1 [file biology-15-01923-s001.zip › Figure S1, S2 legend.pdf]

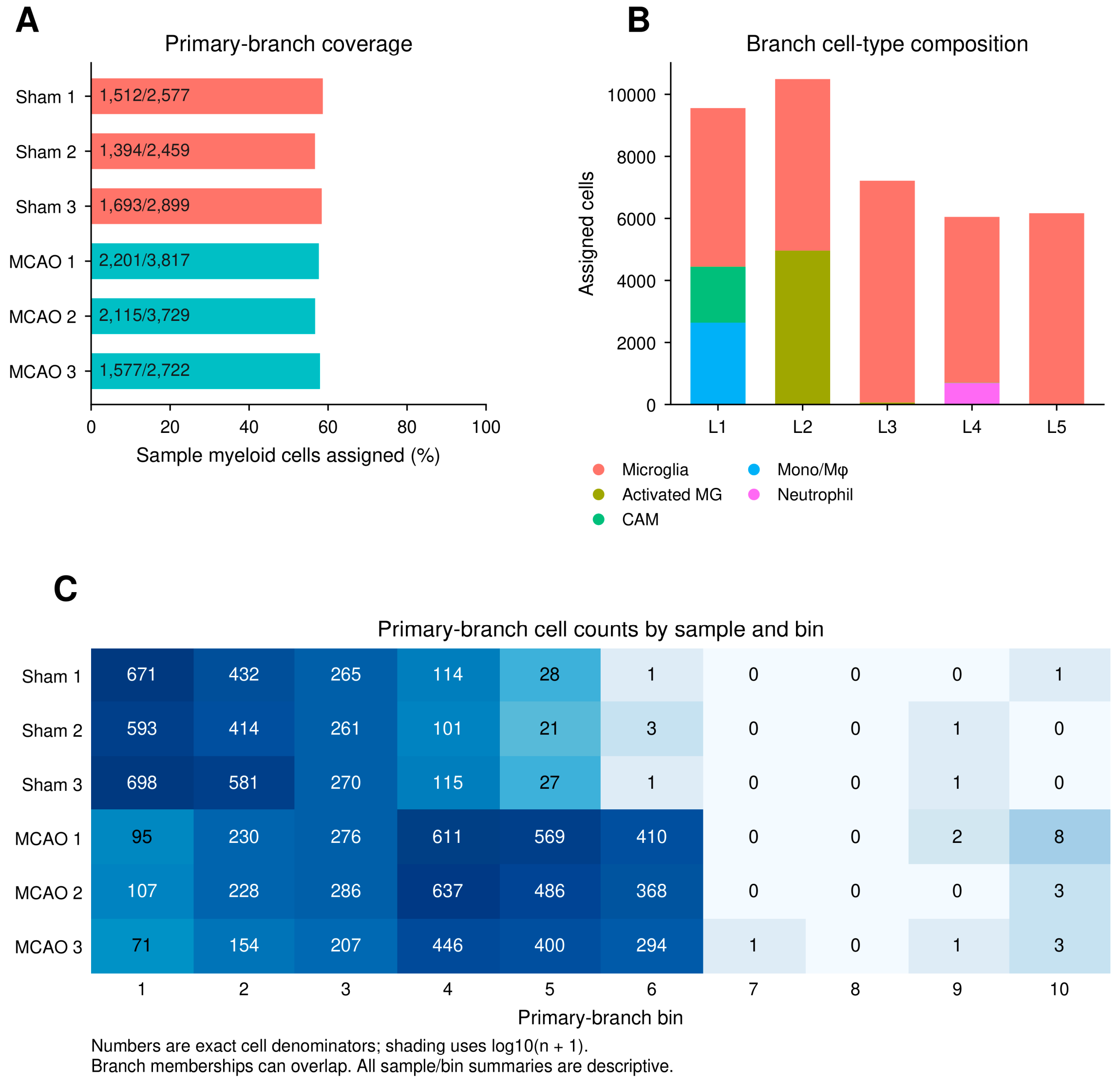

Figure S1. Sample and bin support. (A) Primary-branch coverage for each of the six biological samples; labels give assigned cells divided by all myeloid cells in that sample. (B) Cell-type contributions to the five branches. Memberships overlap and must not be treated as independent populations. (C) Cell counts in every sample/primary-bin combination, with color representing log10(n + 1) and printed values giving the actual n. Zero denotes an empty bin. These are descriptive support displays; they do not increase the number of biological replicates.

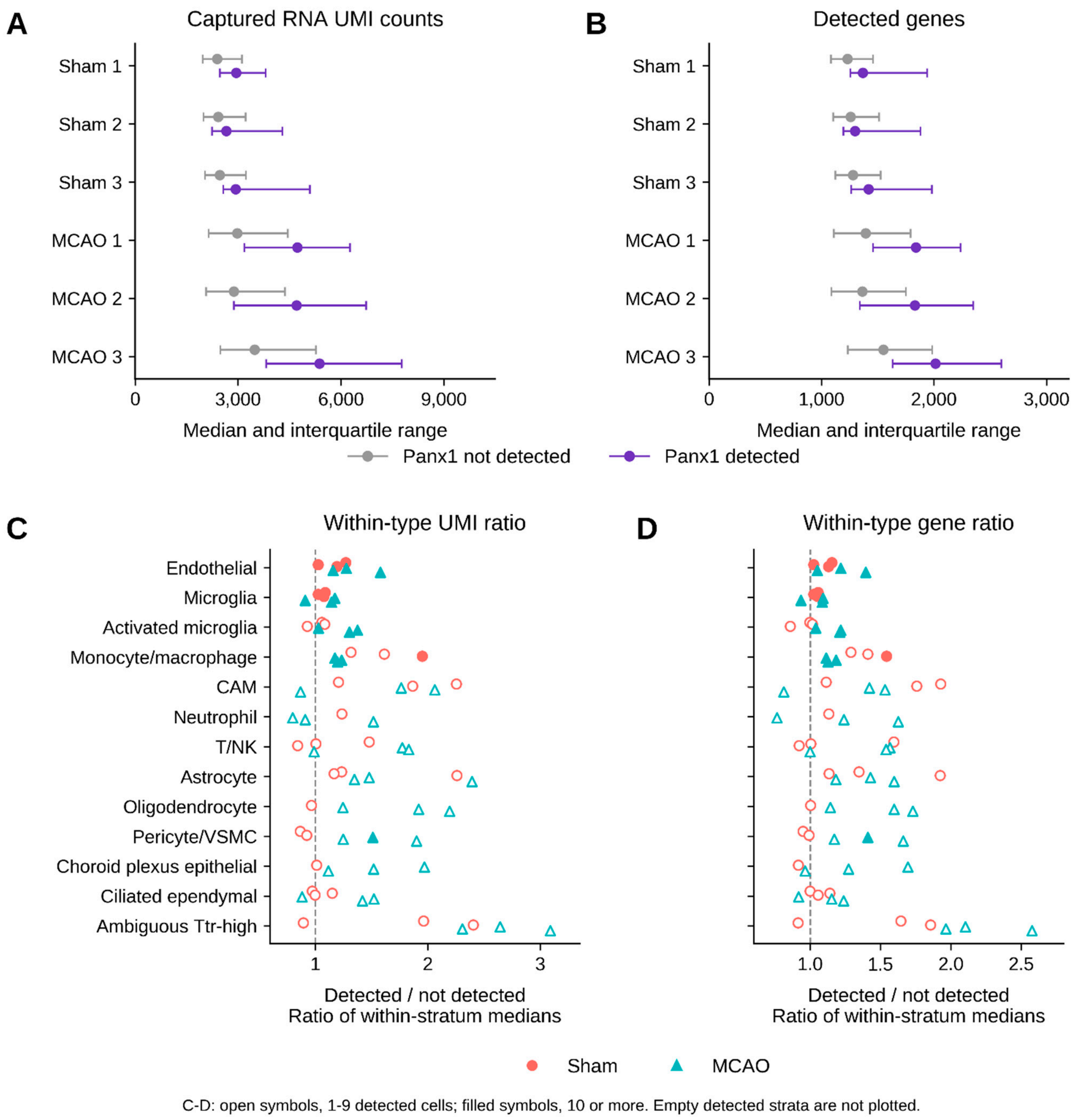

Figure S2. Captured-RNA quality-control summaries by Panx1 transcript-detection status. (A) Recorded RNA-assay UMI totals and (B) detected-gene counts in each of the six biological samples. Points denote within-sample medians; horizontal intervals denote the first to third quartiles of cells, not confidence intervals. Gray denotes Panx1 not detected and purple denotes Panx1 detected (raw RNA UMI > 0). (C) Ratios of median UMI totals and (D) ratios of median detected-gene counts, comparing detected with not-detected cells within the same sample and cell type. Each point is one sample-by-cell-type stratum; the cell-type order is shared between C and D. Circles denote sham and triangles denote MCAO. Open symbols mark 1-9 detected cells, and filled symbols mark at least 10. Seven empty detected strata have

undefined ratios and are not plotted; all 78 strata are retained in Supplementary Data S1. Dashed vertical lines indicate a ratio of one. These summaries are descriptive, do not provide depth-adjusted effects, and do not treat cells or strata from the same sample as independent biological replicates. CAM, CNS-associated macrophage.
