# Supplementary material for: Cellular Context of Panx1 Transcript Detection in Experimental Ischemic Stroke
Source: Biology (Basel). 2026 Oct 23;15(21):1923. doi: 10.3390/biology15211923 (PMC13762306; doi:10.3390/biology15211923)
Supplement: Supplementary file 1 [file biology-15-01923-s001.zip › Supplemental_methods.pdf]

Supplementary Methods

Cellular Context of Panx1 Transcript Detection in Experimental Ischemic Stroke

S1. Datasets and independent units

GSE174574 contributes six mouse brain samples at 24 h: GSM5319987_sham1, GSM5319988_sham2, GSM5319989_sham3, GSM5319990_MCAO1, GSM5319991_MCAO2 and GSM5319992_MCAO3. The suffixes do not define pairs. The final manually annotated atlas contains 58,333 cells, including 18,203 cells in five myeloid classes. Panx1 detection is raw RNA UMI > 0. The biological sample is the unit of condition-level inference; cells, branches and bins are not independent replicates.

GSE58720 contains nine GPL10787 arrays: GSM1418661–GSM1418663 (sham), GSM1418664–GSM1418666 (MCAO), and GSM1418667–GSM1418669 (MCAO plus VEGF). The study-level animal count does not replace the three arrays per group in this reanalysis. The source experiment used 90-min transient MCAO, ventricular recombinant VEGF-A165 administration 3 h after reperfusion, and peri-infarct sampling at 24 h (main-text reference 51).

S2. Implemented single-cell workflow

The supplied final-workflow scripts 03 and 04 create each sample object with min.cells = 3 and min.features = 200, retain 200 ≤ nFeature_RNA ≤ 6000 and percent.mt ≤ 20, and identify mitochondrial genes with ^mt-. Counts labelled before_filter in that workflow are already downstream of object creation. SCTransform uses method = glmGamPoi and regresses percent.mt. Integration uses 3,000 SCT features, reciprocal PCA, 50 per-sample PCs and dimensions 1:30. The integrated object uses 50 PCs, dimensions 1:30 for UMAP/neighbors, and resolutions 0.4, 0.6 and 0.8; manual annotation uses resolution 0.6. Scripts 03 and 04 specify set.seed(1234).

Annotation reviews curated marker-set expression and per-cluster marker summaries, then assigns 27 resolution-0.6 graph-based clusters to 13 annotation classes. The generic seurat_clusters field retains the last generated resolution-0.8 partition and was not used as the resolution-0.6 annotation key. Supplementary Data S4 contains the marker panels, marker expression tables and final mapping. Ambiguous_Ttr_high remains a distinct label. No dedicated computational doublet-removal, ambient-RNA correction or all-gene FindAllMarkers analysis is documented in the supplied final workflow. RNA visualization and per-cell module scores use the RNA LogNormalize data layer. The original NormalizeData call does not explicitly set

scale.factor; an additional undocumented value is not imputed. These processing and annotation limitations are retained in interpretation.

S2.1. Captured-RNA QC by Panx1 detection status

To describe technical factors associated with transcript detection, we summarized the recorded RNA-assay UMI total (nCount_RNA) and detected-gene count (nFeature_RNA) by biological sample, condition, cell type, and Panx1 detection status. Each stratum is reported with its cell count, median, first and third quartiles, mean, and standard deviation in Supplementary Data S1. All 156 sample-by-cell-type-by-status combinations were retained, including empty detected strata with count zero and missing QC summaries. Nonempty strata with fewer than ten cells were flagged rather than excluded. These metrics describe captured RNA in the retained assay, not sequencing-read counts or directly measured cellular RNA content. Pooled summaries and within-sample status comparisons were descriptive; they did not provide depth-adjusted condition effects.

QC summaries were computed from the final cell_metadata_manual_celltypes.csv without filtering additional cells or changing annotations. Quantiles used linear interpolation, equivalent to R quantile type 7, and standard deviations used denominator n - 1; SD is missing when fewer than two cells are present. nCount_RNA and nFeature_RNA were taken from the RNA-assay metadata, not SCT-corrected counts or integrated expression. The summaries refer to the retained assay after the documented object-creation and QC steps, not a reconstruction of all prefilter sequencing reads.

The complete table contains 156 rows (six samples x 13 classes x two detection states). Seven detected strata are empty and retain n = 0 with missing QC values; 51 of the 71 nonempty detected strata contain fewer than ten cells. A separate table compares status-specific medians within each sample and class. The number of strata with a higher median is a descriptive tally; strata share biological samples and are not independent replicates. No cell-level significance test or depth-adjusted regression was added for these QC summaries.

The raw Panx1 UMI histogram contains 57,693 zeros, 619 ones, and 21 twos. All 78 sample-by-class count rows agree with the existing S1 table. An independent recalculation of the 31 locked condition comparisons reproduced the percentage-point effects, Welch intervals, exact permutation P values, and family-specific BH values within numerical precision. This was a reproducibility audit, not an additional family of statistical tests. The QC code and execution information are supplied in S6/reviewer3_qc and S5/R3_QC_AUDIT.json.

S3. Sample-level condition comparisons

For sample s and type t, let D_{st} be the number of Panx1-detected cells and N_{st} the total number of cells of that type. Detection fraction $f_{st} = D_{st}/N_{st}$. Atlas proportions use N_{st} divided by all atlas cells in sample s; myeloid proportions use N_{st} divided by all five myeloid classes in sample s. The effect is $\Delta = 100 \times [\mathrm{mean}(f_{MCAO}) - \mathrm{mean}(f_{Sham})]$, giving equal weight to three samples per condition. Supplementary Data S1 contains all denominators and the 31 planned comparisons (13 detection fractions, 13 atlas proportions and five myeloid proportions).

Cells were first summarized within each biological sample, so the six sample summaries, rather than individual cells, entered the condition test. The two-sided exact permutation P value is the proportion of the 20 possible three-versus-three sample allocations whose absolute mean difference is at least as large as the observed difference. Sample fractions and permutation mean differences are represented as exact rational numbers; no floating-point tolerance is applied. The minimum two-sided P value is 2/20 = 0.10. BH adjustment is performed separately across families of 13, 13 and five. An approximate Welch interval is computed independently: $\Delta \pm t_{0.975,\nu} \times 100 \times \mathrm{sqrt}(s^2_M/3 + s^2_S/3)$, with Welch–Satterthwaite degrees of freedom. The interval is not an inversion of the exact test. A confidence interval excluding zero alongside exact P = 0.10 is therefore not treated as a significant primary test. Leave-one-sample-out effects describe sensitivity; they are not additional confirmatory tests.

S4. Within-condition detection odds ratios

Within each sample, the table entries are a = target detected, b = target not detected, c = reference detected and d = reference not detected. $OR_s = ad/(bc)$. The reference consists of all other 12 atlas types, or the other four myeloid classes. For each condition, $\ell_s = \log(OR_s)$ from the three biological samples is summarized by its arithmetic mean $\bar{\ell}$ and standard deviation s_ℓ. The reported OR is $\exp(\bar{\ell})$, the 95% interval is $\exp[\bar{\ell} \pm t_{0.975,2} \times s_\ell/\sqrt{3}]$, and the two-sided one-sample t test compares $\bar{\ell}$ with zero. These are small-sample, assumption-sensitive summaries of relative detection within a condition, not odds ratios for MCAO versus sham.

If any of the four contingency-table cells is zero in any contributing sample, the aggregate comparison is not estimable (NE). No 0.5 correction is used and no zero-event sample is discarded. Five of the 36 planned summaries are NE. BH and BY adjustments retain planned family sizes of 26 (13 types × two conditions) and ten (five myeloid classes × two conditions); NE results remain missing in the output. The complete sample tables, condition summaries, unadjusted P values and both adjusted values are supplied in S1.

S5. Gene modules and scoring

The following panels were manually specified in the actual scripts. They are descriptive gene sets, not a signature learned from Panx1-detected versus non-detected cells. Main-text references 47–49 provide retrospective biological context, not a recovered original gene-selection log. Per-cell scores average available RNA LogNormalize values without matched-control-gene subtraction. The four first modules below appear in submitted Figure 3E; all seven appear in Figure 4F. Requested genes are listed; Clec7a is absent from the single-cell input.

Single-cell module	Requested genes	Available
Homeostatic microglia	P2ry12, Tmem119, Cx3cr1, Sall1, Hexb	5/5
Activated microglia	Apoe, Lgals3, Lpl, Cst7, Tyrobp, Itgax, Ccl3, Ccl4	8/8
Inflammatory myeloid	Il1b, Tnf, Ccl2, Ccl3, Ccl4, Nfkbia, Ptgs2, S100a8, S100a9	9/9
Phagocytic myeloid	Axl, Clec7a, Trem2, Lpl, Apoe, Ctsb, Ctsd, Lgals3	7/8; Clec7a absent
Chemokine response	Ccl2, Ccl3, Ccl4, Ccl5, Cxcl2, Cxcl10	6/6
Inflammasome like	Nlrp3, Pycard, Casp1, Il18, Il1b, Gsdmd	6/6
Purinergic Panx1 axis	Panx1, P2rx7, P2ry12, P2ry13, Adora2a	5/5

The bulk panel contains Panx1, Apoe, Lgals3, Lyz2, Ccl2, Ccl3, Ccl4, Il1b, Tnf, Nfkbia, S100a8, S100a9, Trem2, Axl, Itgax and Lpl. The six related bulk modules are activated microglial, inflammatory myeloid, phagocytic myeloid, chemokine response, inflammasome-like and purinergic. Their genes match the corresponding rows above, except that Clec7a is not requested in the seven-gene bulk phagocytic panel. There is no bulk homeostatic panel in these seven tests. All requested bulk genes are available. Fifteen of the 16 panel genes occur in at least one of the six related modules; Lyz2 is the exception. The union contains 33 genes. Original script identifiers such as Panx1_associated_myeloid and Purinergic_Panx1_axis are retained in source CSV files solely for traceability; they do not assert Panx1 specificity.

S6. State-space organization and bin support

Slingshot uses the existing UMAP coordinates and resolution-0.6 labels of the 18,203 myeloid cells; no subset embedding is substituted. Candidate root clusters are annotated Microglia with at least 500 cells. They are ordered by lowest MCAO fraction, then highest homeostatic-module mean, then largest cell count. This chooses cluster 2. The condition-informed root limits disease-direction inference. The original call supplies clusterLabels = cluster, reducedDim = UMAP and start.clus = 2; unspecified arguments are recorded separately from explicit choices.

The controlled revision run records Slingshot 2.10.0/princurve 2.1.6 with shrink, reweight and reassign TRUE; extend = y; thresh = 0.001; maxit = 15; stretch = 2; approx_points resolves to 150. These are the installed defaults for that run, not proof of an otherwise unrecovered historical session. No new random seed was imposed for that controlled run. The 10^{-6} value used in the reproduction audit is a comparison tolerance, not the curve-fitting convergence threshold. Stage 3 only redraws locked outputs; it performs no additional fit.

The five cluster paths are 2–19–10–14–9, 2–19–4, 2–19–12, 2–19–16 and 2–19–24. Non-missing memberships number 9,557, 10,492, 7,215, 6,049 and 6,167, respectively. They overlap: 5,787 cells belong to more than one branch, so counts cannot be added as independent populations. The largest branch, Lineage2, is primary. It contains 5,530 Microglia, 4,957 activated microglia, three CNS-associated macrophages and two monocyte/macrophages. Its 7,711 unassigned cells remain gray in the pseudotime display and do not enter primary-bin summaries.

Primary pseudotime is min–max scaled to [0,1], then cut into ten equal-width intervals with right = TRUE and include.lowest = TRUE. Condition/bin means pool cells; the curve x coordinate is the original mean scaled pseudotime of that group. Empty bins are missing, and lines do not bridge them. Open markers flag nonempty groups with fewer than ten cells without changing the data-inclusion rule. Of 60 sample/bin combinations, 15 are empty and 12 contain one to nine cells. The sham Bin6 detection fraction is 1/5 = 20%, with sample denominators of one, three and one. The displays do not test spatial randomness, monotonicity, elapsed time, fate or cross-lineage conversion.

S7. Bulk tissue analysis

GPL10787 annotation maps the processed expression matrix to gene symbols. Among 33,245 annotated probe rows, the highest mean across all nine samples is retained for each symbol, giving 24,163 genes; no tied highest means were present. Probe selection does not maximize a condition contrast. The supplied matrix is used without an additional log transformation. Its upstream logarithm base remains unconfirmed. The source limma column logFC is therefore described as a processed-matrix coefficient and is not exponentiated to produce fold changes. The design is ~0 + group, with MCAO − Sham and MCAO_VEGF − MCAO contrasts. lmFit, contrasts.fit and eBayes generate moderated gene-level statistics. The coefficient interval uses the moderated standard error and corresponding posterior degrees of freedom. BH adjustment covers all 24,163 genes separately for each contrast. S2 includes both full results and the 33-gene union subset; the subset is not readjusted independently.

For gene g and sample s, $zgs = (xgs - mean9(xg))/SD9(xg)$, where SD uses denominator $n - 1$. The panel/module score is the arithmetic mean of the available gene z-scores. Scores are fixed before group-label permutation. Each three-versus-three contrast uses the mean-score difference, all 20 exact label allocations, a separate Welch 95% interval and BH adjustment across seven modules. These highly overlapping modules are not seven independent validations. Two predetermined sensitivities remove Panx1 from the two panels containing it, or standardize across the six sham/MCAO samples. They assess computational dependence on the input definition, not a biological knockout or a separate validation cohort.

S8. Software versions and source files

Software versions / meaning

R 4.3.3; Seurat 5.5.0; SeuratObject 5.4.0; sctransform 0.4.3; Matrix 1.6-5.

R 4.3.3; SingleCellExperiment 1.24.0; slingshot 2.10.0; princurve 2.1.6.

R 4.3.3; limma 3.58.1;

Python 3.12.14; NumPy 2.3.5; pandas 2.2.3; SciPy 1.17.0.

The provenance package distinguishes saved-session evidence, original scripts and later controlled revision runs. It does not equate current installed versions with every historical analysis session. Complete raw bibliometric export, keyword-harmonization dictionary and plotting-parameter records were not available for independent reconstruction during revision. No replacement bibliometric analysis is fabricated.
