# Supplementary material for: Cellular Context of Panx1 Transcript Detection in Experimental Ischemic Stroke
Source: Biology (Basel). 2026 Oct 23;15(21):1923. doi: 10.3390/biology15211923 (PMC13762306; doi:10.3390/biology15211923)
Supplement: Supplementary file 1 [file biology-15-01923-s001.zip › Supplementary Data/Supplementary Data S5/files/022_Figure1A_UMAP_final_celltypes.pdf]

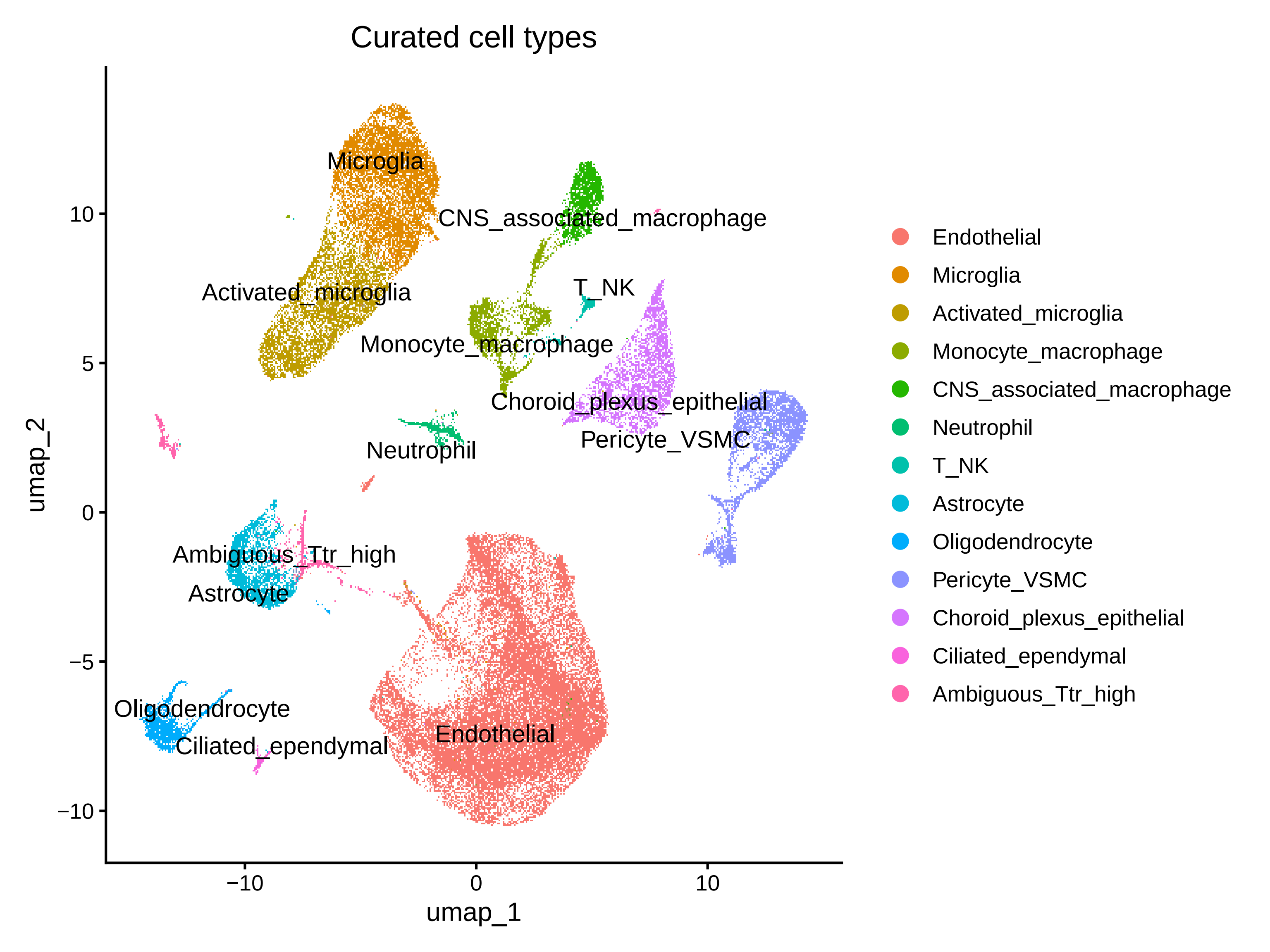
Curated cell types
Microglia
CNS_associated_macrophage
Activated_microglia
T_NK
Monocyte_macrophage
Choroid_plexus_epithelial
Pericyte_VSMC
Neutrophil
Ambiguous_Ttr_high
Astrocyte
Oligodendrocyte
Ciliated_ependymal
Endothelial
umap_2
umap_1
10
5
0
−5
−10
−10
0
10
Endothelial
Microglia
Activated_microglia
Monocyte_macrophage
CNS_associated_macrophage
Neutrophil
T_NK
Astrocyte
Oligodendrocyte
Pericyte_VSMC
Choroid_plexus_epithelial
Ciliated_ependymal
Ambiguous_Ttr_high
