# Supplementary material for: Cellular Context of Panx1 Transcript Detection in Experimental Ischemic Stroke
Source: Biology (Basel). 2026 Oct 23;15(21):1923. doi: 10.3390/biology15211923 (PMC13762306; doi:10.3390/biology15211923)
Supplement: Supplementary file 1 [file biology-15-01923-s001.zip › Supplementary Data/Supplementary Data S5/files/023_Figure1F_marker_dotplot_manual_celltypes.pdf]

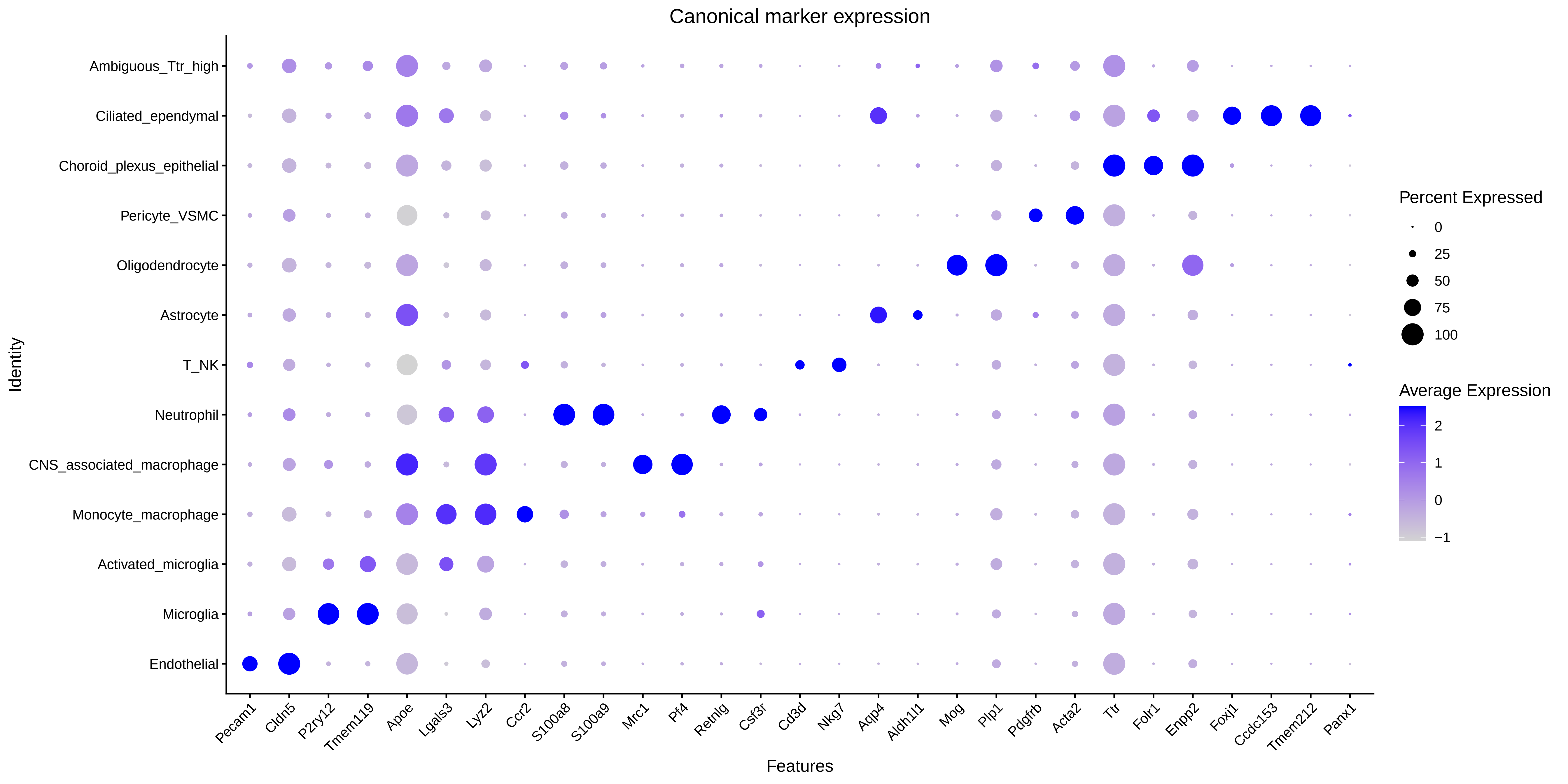

Canonical marker expression
Identity
Ambiguous_Ttr_high
Ciliated_ependymal
Choroid_plexus_epithelial
Pericyte_VSMC
Oligodendrocyte
Astrocyte
T_NK
Neutrophil
CNS_associated_macrophage
Monocyte_macrophage
Activated_microglia
Microglia
Endothelial
Pecam1
Cldn5
P2ry12
Tmem119
Apoe
Lgals3
Lyz2
Ccr2
S100a8
S100a9
Mrc1
Pf4
Retnlg
Csf3r
Cd3d
Nkg7
Aqp4
Aldh1l1
Mog
Plp1
Pdgfrb
Acta2
Ttr
Folr1
Enpp2
Foxj1
Ccdc153
Tmem212
Panx1
Features
Percent Expressed
0
25
50
75
100
Average Expression
2
1
0
−1
