# Supplementary material for: Cellular Context of Panx1 Transcript Detection in Experimental Ischemic Stroke
Source: Biology (Basel). 2026 Oct 23;15(21):1923. doi: 10.3390/biology15211923 (PMC13762306; doi:10.3390/biology15211923)
Supplement: Supplementary file 1 [file biology-15-01923-s001.zip › Supplementary Data/Supplementary Data S5/files/024_Figure2F_myeloid_marker_dotplot.pdf]

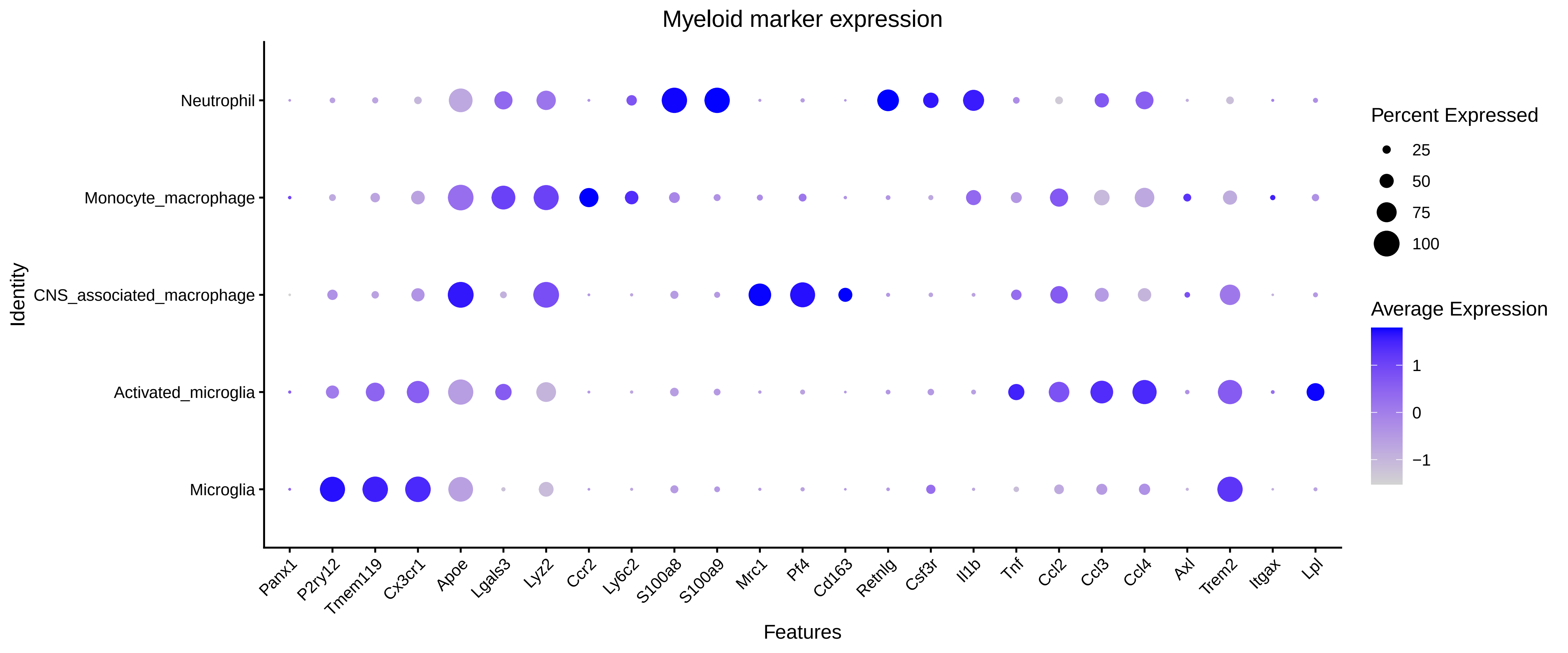
Myeloid marker expression
Neutrophil
Monocyte_macrophage
CNS_associated_macrophage
Activated_microglia
Microglia
Identity
Panx1
P2ry12
Tmem119
Cx3cr1
Apoe
Lgals3
Lyz2
Ccr2
Ly6c2
S100a8
S100a9
Mrc1
Pf4
Cd163
Retnlg
Csf3r
Il1b
Tnf
Ccl2
Ccl3
Ccl4
Axl
Trem2
Itgax
Lpl
Features
Percent Expressed
25
50
75
100
Average Expression
1
0
−1
